# CUX1, A Controversial Player in Tumor Development

**DOI:** 10.3389/fonc.2020.00738

**Published:** 2020-05-29

**Authors:** Ning Liu, Qiliang Sun, Long Wan, Xuan Wang, Yu Feng, Judong Luo, Hailong Wu

**Affiliations:** ^1^Department of Clinical Oncology, Taian City Central Hospital, Tai'an, China; ^2^Department of Respiratory Medicine, Taian City Central Hospital, Tai'an, China; ^3^Department of Liver Diseases, Central Laboratory, Institute of Clinical Immunology, ShuGuang Hospital Affiliated to Shanghai University of Chinese Traditional Medicine, Shanghai, China; ^4^Department of General Surgery, Shuguang Hospital, Shanghai University of Traditional Chinese Medicine, Shanghai, China; ^5^Department of Radiation Oncology, The Affiliated Changzhou No.2 People's Hospital of Nanjing Medical University, Changzhou, China; ^6^Shanghai University of Medicine & Health Sciences Affiliated Zhoupu Hospital, Shanghai, China; ^7^Collaborative Innovation Center for Biomedicine, Shanghai University of Medicine & Health Sciences, Shanghai, China; ^8^Shanghai Key Laboratory of Molecular Imaging, Shanghai University of Medicine & Health Sciences, Shanghai, China

**Keywords:** CUX1, haploinsufficient tumor suppressor, tumor progression, DNA damage, KRAS mutation

## Abstract

CUX1 belongs to the homeodomain transcription factor family and is evolutionarily and functionally conserved from *Drosophila* to humans. In addition to the involvement in various physiological events including tissue development, cell proliferation, differentiation and migration, and DNA damage response, CUX1 has been implicated in tumorigenesis. Interestingly, *CUX1* has been recently recognized as a haploinsufficient tumor suppressor, which is paradoxically overexpressed in tumor cells. While loss of heterozygosity and/or mutations of *CUX1* have been frequently detected in many types of cancers, genomic amplification, and overexpression of *CUX1* have also been reported in cancer tissues and are correlated with higher tumor grade and poor prognosis. Therefore, deciphering the roles of different CUX1 isoforms and in different tumor stages is required to establish a CUX1-based therapeutic strategy for cancer treatment.

## Introduction

*CUX1* is previously called CDP (CCAAT displacement protein), Cut-like 1 (CUTL1), or Cut [reviewed in Sansregret and Nepveu (1) and Hulea and Nepveu (2)]. The term “cut” was derived from a *Drosophila* mutant with the “cut wing” phenotype (3, 4). In 2007, the Human Genome Organization proposed to change the gene root of Cut-like# (CUTL#) to CUT#. Therefore, *CUX1* (human gene), *Cux1* (mouse gene), and CUX1 (protein) are the simplified nomenclature. *CUX1* belongs to the homeodomain (HD) transcription factor family, which was first identified as a sea urchin transcription repressor of the sperm H2B gene by binding to promoter element–CCAAT and competing the binding of other transcriptional activators (5). However, some other studies have shown that CUX1 may also function as either a transcriptional repressor or an activator in a promoter-dependent manner (6–8). *CUX1* is present in all metazoans and evolutionarily and functionally conserved from *Drosophila* to humans, because ectopic expression of human or mouse CUX1 can rescue a wing scalloping mutant phenotype caused by loss of *cut* (the *Drosophila* ortholog of *CUX1*) expression along the prospective wing margins in *Drosophila* (9). The human *CUX1* is at least 340 kb in length and located on the chromosome 7q22 (10). As a transcription factor, CUX1 has been implicated in cell proliferation, differentiation, and migration in various tissues and organs (1, 11–13) [reviewed in Vadnais et al. (8)]. Ectopic overexpression of *Cux1* leads to multiorgan hyperplasia in a transgenic mouse model (14, 15). Two distinct *Cux1* knockout mouse lines exhibit various phenotypes such as high postnatal lethality, growth retardation, nearly complete hair loss, severely reduced male fertility due to behavioral reasons, cachexia due to muscle wasting and loss of body fat, thin and flaky bones, and abnormal hematopoiesis (16–18) [reviewed in Sansregret and Nepveu (1)]. In addition to its physiological functions, emerging evidence has shown the involvement of CUX1 in tumorigenesis [reviewed in Hulea and Nepveu (2), Liu et al. (19), and Ramdzan and Nepveu (20)], but the exact roles of CUX1 in tumor development are still under debate. In this review, we introduce the protein structures and isoforms of CUX1, describe the various biological processes in which they are involved, summarize the role of CUX1 in tumor development and progression, and discuss the possible explanations related to the paradoxical roles of CUX1 in tumor development.

## Structures and Isoforms of CUX1

**Figure 1:**
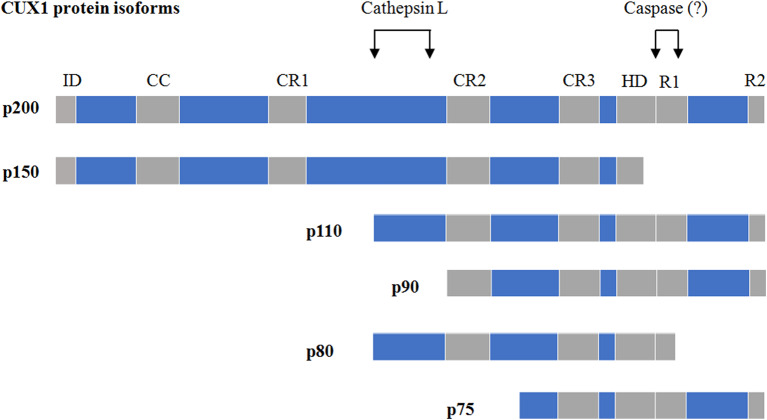
The isoforms of CUX1 and proteinases responsible for their proteolysis. ID, autoinhibitory domain; CR, cut repeats; HD, homeodomain; R1/R2, two active repression domains. The figure is partly modified from Vadnais et al. (8).

As a transcriptional factor, CUX1 contains four DNA-binding domains including three Cut repeats (CR1, CR2, and CR3) and one HD (21) (Figure 1). In addition, CUX1 also carries one autoinhibitory domain (ID) at its N-terminus (22) and two active repression domains (R1 and R2) at its C-terminus (23) (Figure 1). CUX1 protein possesses multiple isoforms generated from either proteolysis of full-length CUX1 or alternative transcription initiation of *CUX1* gene [reviewed in Sansregret and Nepveu (1) and Hulea and Nepveu (2)]. According to their apparent molecular weight, those CUX1 isoforms were named p200 (full-length CUX1), p150, p110, p90, p80, and p75. Among them, the p150, p110, p90, and p80 are the products of proteolysis of full-length CUX1. The generation of p110 and p90 was mediated by a nuclear cathepsin-L, which removes the N-terminal half of CUX1 (24, 25) (Figure 1), whereas the isoform p80 is a result of two proteolytic events catalyzed by the nuclear cathepsin-L and an unknown caspase at the N- and C-terminal sides, respectively (26), leading to a removal of both the N-terminal half and a region at the C-terminus (Figure 1). Notably, apoptosis onset is not required for such a caspase-mediated p80 processing (26), suggesting the existence of apoptosis-independent role of caspases. The isoform p150 is a proteolytic product of CUX1 at the C-terminal region, but which protease is responsible for p150 processing remains unknown (27, 28). The isoform p75 is encoded by a short *CUX1* transcript, which is generated from an alternative transcriptional initiation site within the intron 20 (29). In addition, neutrophil elastase has also been reported to proteolytically process full-length CUX1 to generate short CUX1 isoforms (30, 31).

The DNA-binding patterns and/or dynamics of CUX1 isoforms are largely determined by which DNA-binding domains are present in them. Although the full-length CUX1 contains all the four DNA-binding domains (three CRs and one HD), it binds to DNA at the –CCAAT motif in a rapid but transient manner and exclusively functions as a transcriptional repressor (32). The isoform p150 with an impaired HD is incapable of binding to DNA and functions as a dominant-negative isoform in the lactating mammary gland (28), whereas the isoforms p110, p90, and p75 with the removal of the N-terminal ID and the CR1 could slowly but stably bind to DNA at the ATCRAT motifs and function as either a repressor or an activator in a promoter-dependent manner (6–8, 33–35).

## The Involvement of CUX1 in Various Biological Processes

Physiologically, CUX1 has been reported to play important roles in tissue development, cell migration, proliferation and differentiation, and DNA damage repair.

### Roles of CUX1 in the Development of Nervous System

CUX1 expression is detectable in layers II–V in human developing neocortices in the fetal period and disappeared until 3 months of age after birth, suggesting the role of CUX1 in the development of human neocortex (36). Analyses of loss- and gain-of-function of *cut* mutants in *Drosophila* have revealed the roles of *cut* (the *Drosophila* ortholog of *CUX1*) in the peripheral nervous system. There are two types of anatomically distinguishable sensory organs, external sensory (es) organs, and internal (chordotonal) sensory organs. In *Drosophila, cut* is exclusively expressed in cells of the es organ but repressed in cells of the chordotonal organ (37, 38). Therefore, the lethal *cut* mutants exhibit the transformation of es organs into chordotonal organs (37, 39, 40), whereas forced *cut* overexpression in *Drosophila* embryos resulted in the conversion of chordotonal organs into es organs (41). Moreover, the level of Cut is a determinant of the distinct dendrite branching patterns of dendritic arborization (da) sensory in *Drosophila* (42). Cux1 and Cux2, a homolog protein of Cux1, have been shown to stimulate dendrite branching, spine development, and synapse formation in layers II–III neurons of the cerebral cortex (43, 44). However, an *in vitro* study showed opposite results indicating that Cux1 suppresses dendritogenesis of neuronal cells (45). Therefore, additional studies are required for clarifying the role of Cux1 in the development of nervous system.

### Roles of CUX1 in Cell Proliferation, Differentiation, and Migration

**Figure 2:**
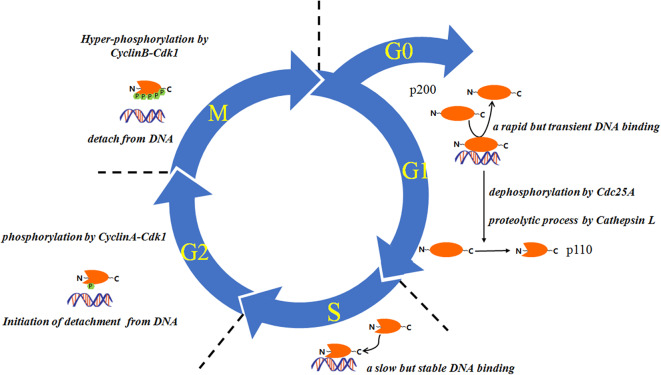
The proteolysis and DNA binding of CUX1 are regulated in a cell cycle–dependent manner. This figure is partly modified from Vanden Heuvel et al. (14).

A number of studies have indicated that the expression and/or the DNA-binding dynamics of CUX1 are in a cell cycle–dependent manner (Figure 2). For example, the expression of histone nuclear factor D (HiNF-D), which includes CUX1 as its DNA-binding partner, was upregulated in S-phase in normal cells (46–50); the CUX1-DNA binding was undetectable in G0 and early G1 phase, became detectable in the late G1 phase, and peaked in S phase (11). This dynamic change of CUX1-DNA binding is attributed to at least two posttranslational modifications, Cdc25A-mediated dephosphorylation at the CUX1 HD domain, and cathepsin L–mediated proteolytic cleavage to generate p110 CUX1 (11, 24, 33); in G2 phase, the binding of CUX1-DNA was attenuated due to CyclinA-Cdk1 mediated phosphorylation of CUX1 (51). In addition, Alain Nepveu group also demonstrated that the cyclin B/CDK1–mediated hyperphosphorylation of CUX1 could reset CUX1 DNA-binding activity to the zero level at each cell division (52). On the other hand, a subset of the downstream target genes of CUX1 has been reported to play a role in cell cycle progression (6, 7, 34, 53). These studies together suggest the involvement of CUX1 in cell cycle regulation and cell proliferation. In line with these findings, cells with p110 CUX1 overexpression showed accelerated entry into S phase and cell proliferation, whereas mouse embryo fibroblasts derived from *Cux1*^z/z^ mutant mice showed an extended G1 phase and retarded cell proliferation (54). Moreover, *Cux1* transgenic mice displayed organomegaly and multiorgan hyperplasia (14). All these *in vitro* and *in vivo* findings indicate the implication of CUX1 in cell proliferation.

Early studies have demonstrated that *Cux1* is exclusively expressed in undifferentiated cells (21, 55–58), suggesting the role of CUX1 in cell differentiation. In both mice and humans, *Cux1* expression is high in long-term hematopoietic stem cell (LT-HSC) but low in short-term hematopoietic stem cell (ST-HSC) and myeloid progenitors, and *in vivo Cux1* knockdown led to expansion of myeloid and ST-HSC, suggesting that *Cux1* may be essential for maintaining HSC quiescence, suppressing HSC proliferation and self-renewal, and regulating lineage specification and differentiation (59). *Cux1* nonfunctional mutant mice on inbred backgrounds die shortly after birth due to retarded differentiation of the lung epithelia, and the survival outbred *Cux1* nonfunctional mutant mice exhibit an abnormal pelage because of disrupted hair follicle morphogenesis, suggesting that *Cux1* is essential for the differentiation of epithelia in lung and hair follicle (18).

In addition to cell proliferation and differentiation, some recent studies have disclosed the involvement of Cux1 in cell migration and invasion. A high-throughput RNAi screening demonstrated that *Cux1* knockdown led to impaired cell migration and invasion in NIH-3T3 and a series of human cancer cell lines (12). Correspondingly, MEF cells (MEFs) derived from *Cux1* knockout mice are defective in migration and invasion compared to MEFs derived from wild-type mice (13). Interestingly, the migration defect in *Cux1* knockout MEFs can be completely rescued by p110 CUX1 but partially rescued by p200 CUX1 (full-length CUX1) (35). Moreover, stimulated or inhibited proteolytic processing of p200 CUX1 can, respectively, enhance or decrease cell migration, suggesting that CUX1-mediated cell migration may be attributed to its proteolytic products (13, 35).

### Relationship Between CUX1 and DNA Damage Repair

Genomic integrity is critical for proper cellular function and faithful transmission of genetic information to progeny. In addition to implicating in cell proliferation, differentiation, and migration, accumulating evidence has indicated the involvement of CUX1 in DNA damage response (DDR). According to a genome-wide location analysis of p110 CUX1, 18 DDR-related genes, including *ATM* (ATM serine/threonine kinase) and *ATR* (ATR serine/threonine kinase), were suggested to be the putative targets of p110 CUX1 (6, 7). A subsequent study further confirmed the direct transcriptional regulation of CUX1 on those DDR genes and suggested that CUX1 is required for ATM- and/or ATR-mediated DNA repair in response to DNA damages induced by ionizing radiation (IR) and/or ultraviolet (UV), respectively (6). In addition to direct transcriptional regulation of DDR-related genes, CUX1 was reported to function as an accessory factor to promote DNA damage repair independent of its transcriptional activity (60–64). By directly interacting with 8-oxoguanine DNA glycosylase 1 (OGG1), CUX1 can stimulate the DNA binding, Schiff-base formation, glycosylase, and apurinic/apyrimidinic (AP)-lyase activities of OGG1 to enhance the removal of ROS-induced DNA adducts, 7,8-dihydro-8-oxoguanine (8-oxoG) (63). The direct activity of DNA damage repair of CUX1 is mainly attributed to its CUT domains because a CUX1 recombinant protein containing only CUT domains 1 and 2 is sufficient to accelerate DNA damage repair (62). More interestingly, in line with this finding, some other CUT domain proteins have also shown to be directly involved in base excision repair (65, 66), suggesting that the CUT domain may serve as a therapeutic target of tumor in response to DNA damage.

## Roles of CUX1 in Tumor Development

In addition to its physiological functions, CUX1 has been implicated in tumor development in many species including *Drosophila*, mouse, and humans. But whether CUX1 functions as an oncogene or tumor suppressor is still under debate, because the results of CUX1 studies on tumor development are controversial.

### CUX1 Serves as an Oncogene

There are several lines of evidence indicating the oncogenic role of CUX1. First, elevated expression of CUX1 has been observed in many types of cancers, including colorectal cancer (67), multiple myeloma (68), uterine leiomyomas (69), high-grade breast cancer (12), pancreatic cancer (70), melanoma (71), and glioma (72); second, CUX1 expression is positively associated with poor prognosis in glioma, glioblastoma, colorectal cancer, breast cancer, and pancreatic cancer (12, 61, 67, 72); third, mouse mammary tumor virus (MMTV) p200, p110, and p75 CUX1 transgenic mice develop late-onset mammary carcinoma (64, 73); fourth, active *Kras* mutations, such as KRAS^G12D^ and KRAS^Q61L^, have been observed in mammary carcinomas from MMTV-p200 CUX1 transgenic mice, and CUX1 can cooperate with KRAS^G12V^, an active *Kras* mutant, to promote lung tumor formation *in vivo* (64); fifth, CUX1 is a transcriptional target downstream of the transforming growth factor β and/or PI3K-AKT signalings and contributes to enhanced proliferation, migration/invasion, and reduced apoptosis in tumor cells (12, 70). More interestingly, a very recent study has demonstrated that *CUX1* can generate a circular RNA (*circ-CUX1*) to promote tumor progression in neuroblastoma (NB) (74). This *circ-CUX1* carries exon 2 and partial intron 2 of *CUX1* and is up-regulated in NB tissues and cell lines. The levels of *circ-CUX1* negatively associate with the survival probability in NB patients. *Circ-CUX1* can directly interact with EWSR1 and facilitate EWSR1-MAZ interaction, resulting in transactivation of MAZ and transcriptional alteration of *CUX1* and other genes associated with tumor progression (74).

The mechanisms by which CUX1 promotes tumor development have been investigated in many types of cancers especially in breast cancer and pancreatic cancer. For example, by cooperating with GLIS1, CUX1 can stimulate autocrine activation of the Wnt/β-catenin pathway to enhance cell migration and invasion in breast cancer (75). CUX1 stimulates migration and invasion by transcriptionally activating or repressing a series of target genes related to cell motility, including activating snail and slug and repressing E-cadherin, in breast cancer cells (13). CUX1 stabilizes Src and in turn activates its downstream signaling molecules such as RhoA, Rac1, Cdc42, and ROCK by transcriptionally upregulating C-terminal Src kinase (Csk) in pancreatic cancer (76). CUX1 can transcriptionally upregulate WNT5A and GRIA3 to reduce apoptosis and promote proliferation, migration, and invasiveness in pancreatic cancer (77, 78). The p110 CUX1 activates a transcriptional program that reinforces the spindle assembly checkpoint and delays mitosis until extranumerary centrosomes have clustered to two poles, thereby enabling bipolar mitosis and survival of tetraploid cells (53). CUX1 was reported to promote the aggressiveness of pancreatic neuroendocrine tumor partly through modulating MMP9 expression (79). In addition, CUX1 was enriched in tumor-associated macrophages (TAMs) and interacted with nuclear factor κB (NF-κB) p65 to attenuate the activation of NF-κB signaling, leading to a decrease in T-cell attraction and an increase in angiogenesis in pancreatic cancer (80).

### CUX1 Acts as a Tumor Suppressor

Although some previous studies have indicated the oncogenic role of CUX1 in tumor progression, other studies also exhibit substantial evidence to support CUX1 as an important tumor suppressor in many types of cancers.

The evidence of *CUX1* as a tumor suppressor first emerged from cytogenetic studies showing 7q^−^ (deletions within the long arm of chromosome 7) in many types of cancers, including myeloid leukemia, pancreatic carcinoma, kidney carcinoma, colon carcinoma, ovarian carcinoma, lung carcinoma, head-and-neck carcinoma, cholangiocarcinoma, uterine leiomyoma, and breast cancer (81–88). Next, loss of heterozygosity (LOH) analyses confirmed LOH at 7q22 in a subset of breast cancer, uterine leiomyoma, and ovarian cancer (88–91), suggesting that genes in this region including *CUX1* may function as tumor suppressors. Furthermore, *CUX1* was late identified as a haploinsufficient tumor suppressor in acute myeloid leukemia, because RNAi-mediated *cut* (the *Drosophila* ortholog of *CUX1*) knockdown led to the development of melanotic pseudotumors in a *Drosophila* tumor model (92). More importantly, a comprehensive study by interrogating total 7,651 genome sequences derived from 28 tumor types revealed nonsense and frameshift mutations in *CUX1* in 1–5% of tumors and found that CUX1 deficiency can lead to activation of the pro-oncogenic PI3K-AKT signaling (93). In addition, CUX1 has been shown to negatively regulate invasion in castrate-resistant prostate cancer (94) and multidrug resistance in gastric cancer (95). It is worth mentioning that, although loss and/or inactivation of a *CUX1* allele have been documented in many studies, there is so far no case of a tumor where both alleles have been lost or inactivated, suggesting the coexistence of an inactivated and an activated *CUX1* alleles in tumor cells.

### Possible Explanations on the Opposite Roles of CUX1 in Tumor Progression

It seems paradoxical that *CUX1* possesses both oncogenic and tumor-suppressive features. One possible explanation comes from the protective role of CUX1 in DNA damage repair, because the machinery of DNA damage repair is a double-edge sword in tumor initiation and progression. On the one hand, an effective DNA-repair machinery is required to prevent accumulation of DNA lesions, genomic instability, and subsequent malignant transformation in normal cells, suggesting the suppressive roles of DNA repair in tumor initiation (96). On the other hand, a basal repair activity is also essential for tumor cells to avoid DNA damage–induced cell death (97). Given the involvement of CUX1 in both exogenous DNA damage (induced by temozolomide, H_2_O_2_, UV, and IR) and endogenous DNA damage (induced by intracellular ROS) (6, 7, 61, 63, 64), it is conceivable that CUX1 may, respectively, function as a tumor suppressor or an oncogene in the stages of tumor initiation or progression.

The second explanation rises from the existence of various CUX1 isoforms and their divergent transcriptional activities. By alternative transcriptional initiation and/or proteolytic processing, CUX1 generates several short isoforms that possess distinct DNA-binding capacity and transcriptional activities compared to the full-length CUX1 (p200 CUX1). p200 CUX1 binds to DNA in a rapid but transient manner and exclusively functions as a transcriptional repressor through either competition occupancy for CCAAT or Sp1 binding sites or active repression by the recruitment of histone deacetylases (HDACs) (23, 98). However, in contrast to full-length CUX1, the short isoforms can bind to DNA stably and function as either transcriptional repressors or activators in a promote-specific manner (33–35). Because most of the short isoforms are derived from the full-length *CUX1* transcript, RNAi approach is not able to specifically knock down indicated isoforms, which makes RNAi not suitable to determine the roles of full-length CUX1 and its isoforms in tumor progression. So far, from the results of overexpression studies, it is clear that the CUX1 short isoforms may mainly function as oncogenes. For example, MMTV-p110, p75 transgenic mice developed mammary tumors after a long latency period, and genes involved in Wnt/β-catenin signaling were directly regulated by those short CUX1 isoforms (73). Stimulation or inhibition of the proteolytic processing of p200 CUX1 toward p110 CUX1 can, respectively, enhance or attenuate cell migration, suggesting that the p110 CUX1 but not the p200 CUX1 plays a major role in promoting cell migration (13). p200 CUX1 is proteolytically processed into p110 CUX1 by a nuclear cathepsin L at the G_1_/S transition, and forced overexpression of p110 CUX1 stimulates cell proliferation (24, 54). Moreover, the nuclear accumulation and activity of cathepsin L were increased in many transformed cells in parallel with augmented CUX1 processing, and the cell-permeable but not the non–cell-permeable inhibitors of cathepsin L delay the entry into S phase and proliferation in transformed cells (99). These findings suggest that the short CUX1 isoforms contribute to its oncogenic role in tumor progression. However, the exact role of p200 CUX1 in tumor progression is still under debate. A previous study had shown that MMTV-p200 mice developed mammary tumors with a slightly higher penetrance than the MMTV-p75 or p110 CUX1 mice by promoting faster DNA repair, thereby allowing transformed cells to avoid senescence and continue to proliferate (64), suggesting the oncogenic role of p200 CUX1. But recent findings showing that many types of tumors possess nonsense or frameshift mutations of CUX1 paradoxically suggest that p200 CUX1 may function as a tumor suppressor (93). Very recently, by employing K562 cells, which predominantly express the p200 CUX1, Arthur et al. (100) have demonstrated that p200 CUX1 binds distal *cis*-regulatory elements associated with gene activation, but the coexistence of p200 CUX1 and its short isoforms in many other cancer cells is still a bottleneck in the process to disclose the exact roles of them in tumor development. Therefore, more precise and sophisticated studies, for example, by employing CRISPR/Cas9 genome editing approach to establish either p200 CUX1 unprocessable or p200 CUX1 null mutants, are required to define the roles of p200 CUX1 and its isoforms in tumor development.

The third explanation derives from the presence of CUX1 in both tumor cells and TAMs and the inhibitory effects of CUX1 on the NF-κB signaling. The Michl P group has demonstrated the overexpression of CUX1 in both tumor cells and TAMs in pancreatic cancer (70, 80). They demonstrated that the full-length CUX1 interacts with NF-κB p65 and HDAC1 to form a protein trimer to repress NF-κB signaling in TAMs, resulting in inhibition of M1 polarization and enhanced angiogenesis and tumor progression (80). While inhibition on NF-κB signaling in TAMs promotes tumor progression, aberrant and constitutive activation NF-κB signaling is frequent in tumoral cells and shows positive effects on tumor progression (101, 102). Therefore, if CUX1 can inhibit NF-κB signaling in both tumor cells and TAMs, CUX1-mediated inhibition on NF-κB signaling in tumor cells or TAMs may, respectively, suppress or promote tumor development, which may contribute to the paradoxical roles of CUX1 to tumor development.

## Summary

As an evolutionarily conserved transcription factor, CUX1 is expressed in almost all metazoans. Because of the proteolytic processing or alternative transcriptional initiation, CUX1 possesses multiple isoforms with differential DNA-binding capacity and transcriptional activity. The full-length CUX1 binds to DNA in a rapid but transient manner to exclusively repress gene expression by either passively occupying the binding sites of transcriptional activators or actively recruiting HDACs to achieve epigenetic silencing. CUX1 is physiologically implicated in tissue development, cell proliferation, differentiation and migration, and DNA damage repair. The inbred *Cux1*^−/−^ or inactive mice are postnatal lethal due to retarded differentiation of the lung epithelia, and the survival outbred *Cux1*^−/−^ mice exhibit an abnormal pelage because of disrupted hair follicle morphogenesis. The pathological involvement of CUX1 in tumorigenesis is complicated. Both sides of evidence, respectively, support the tumor suppressive or oncogenic roles of CUX1 in tumor development and progression. So far, the short isoforms of CUX1, such as p110 and p75 CUX1, seem to carry oncogenic features, while the exact role of full-length CUX1 in tumor progression remains elusive. Therefore, further studies specifically targeting full-length CUX1 or short isoforms are required to decipher the role of CUX1 in tumor progression.

## Author Contributions

NL, QS, LW, XW, and YF performed extensive literature search and discussion. NL drafted the manuscript. JL and HW edited the manuscript.

## Conflict of Interest

The authors declare that the research was conducted in the absence of any commercial or financial relationships that could be construed as a potential conflict of interest.
